# Biochemical metabolic enhancement acting as a dominant driver in intra-leaf CO_2_ diffusional response to soil nitrogen supplying in Soybean

**DOI:** 10.1371/journal.pone.0340250

**Published:** 2026-01-30

**Authors:** Qihui Zuo, Siyu Tan, Lina Gao, Xueer Wang, Jian Zhang, Fenwu Liu, Kai Zhu

**Affiliations:** 1 College of Resources and Environment, Shanxi Agricultural University, Taigu, China; 2 State Key Laboratory of Sustainable Dryland Agriculture (in preparation), Shanxi Agricultural University, Taiyuan, China; 3 College of Veterinary Medicine, Shanxi Agricultural University, Taigu, China; Guizhou University, CHINA

## Abstract

Biochemical metabolism and anatomical structure within leaf tissues have been proposed as the two principal mechanisms underpinning the rapid responsiveness of mesophyll conductance (*g*_m_) to environmental perturbations; nevertheless, empirical evidence distinguishing which of these factors acts as the dominant driver remains scarce. The response of intra-leaf CO_2_ diffusion conductance including *g*_m_ and stomatal conductance (*g*_sc_) to soil nitrogen (N) change in soybean was systematically quantified in leaf biochemical and structural characteristics. Our data revealed that (i) soil N made a positive effect on intra-leaf CO_2_ diffusion and carbon assimilation that *g*_m_ and *A*_n_ (net photosynthetic rate) exhibited a significant positive response to increasing N supplying from 7.5 to 15.0 g urea m^-2^ while *g*_sc_ showed no significant N-dependence. (ii) The enhanced intra-leaf CO_2_ diffusion capacity induced by N application was principally attributable to the increase in *g*_m_.(iii) The enhancement of biochemical metabolism rather than the modifications in the leaf anatomical structure constituted the predominant mechanism by which N supplementation facilitated CO_2_ diffusion and carbon assimilation in soybean. (iv)Furthermore, the improvement in water use efficiency (WUE) appeared to be more closely linked to aquaporin-mediated water relations, as supported by subsequent correlation analyses.These findings will advance our understanding of the key drivers that shape *g*_m_ responsiveness to abrupt environmental variations.

## Introduction

Like drought in soil, atmospheric nitrogen (N) deposition has become another significant ecological factor affecting plant growth and metabolism as global climate change intensifies.Global warming, by altering atmospheric circulation and hydrological processes, will lead to changes in annual precipitation and its seasonal distribution at both global and regional scales. This can further result in more frequent, prolonged, and intensified droughts in some regions [1]. Model simulations indicate that from 1986 to 2050, the spatial extent of nitrogen deposition will continue to expand globally, with a gradually increasing trend in deposition rates [2]. It is projected that by 2030, global nitrogen deposition will reach 105 Tg per year [3]. Excessive N deposition can severely weaken the photosynthetic carbon assimilation process in plants.As an important indicator of photosynthetic carbon assimilation, intra-leaf CO_2_ diffusion conductance, including CO_2_ stomatal conductance (*g*_sc_) and mesophyll conductance (*g*_m_) can significantly respond to N availability [4].The Loess Plateau region is located in arid and semiarid regions of central and western China and includes Shanxi, Shaanxi, Gansu, and Ningxia, which are important areas of agricultural production in China. With global climate change, the amount of atmospheric N deposition in this region is gradually increasing to 38.9 kg N ha^-1^ a^-1^ [5], which results in interactive stresses on plant growth and photosynthetic primary productivity, especially *g*_sc_ and *g*_m_, in the local arid climate socially.

There are multiple mechanisms through which N regulates photosynthetic carbon assimilation, with leaf structure and biochemical metabolism being regarded as the two predominant factors underlying variations in intra-leaf CO_2_ diffusion conductance [4,6,7]. Nitrogen enhances leaf structural and biochemical properties, inducing alterations in *g*_sc_ and *g*_m_ parameters, Consequently, substantially boosting the rate of photosynthetic CO₂ assimilation in plants [8,9]. Through its effect on mesophyll cell arrangement (enhanced to a certain extent by nitrogen), the surface areas of mesophyll cells and chloroplasts exposed to the intercellular space per unit leaf area (*S*_m_ and *S*_c_) are increased [9], Driving the directional repositioning of chloroplasts to alleviate photo-oxidative damage to the photosynthetic apparatus [10]. In addition, nitrogen can also increase the stomatal opening (*SS*) [11]; improve the activity of aquaporins (AQPs), carbonic anhydrase (CA) and Rubisco (ribulose-1,5-bisphosphate carboxylase); and reduce the content of abscisic acid (ABA), significantly promoting stomatal and mesophyll CO_2_ diffusion. However, excessive nitrogen has been found to increase the ABA content and reduce AQPs, CA, and Rubisco activities [6,9,12–14], thereby reducing stomatal opening and CO_2_ transmembrane transport to weaken photosynthetic carbon assimilation. Compared with the dominant contribution of AQPs in regulating *g*_sc_ and *g*_m_, the role of CA in regulating the intra-leaf CO_2_ diffusion capacity is controversial. Few scholars have maintained that CA has little effect on *g*_m_ [15,16], but more studies have resolutely supported its positive role in the increase in *g*_m_, suggesting that it accelerates the metabolism of CO₂ into bicarbonate ions and has a significant biochemical effect on CO_2_ diffusion [17–21]. Our previous studies revealed the positive effects of AQPs and CA on *g*_m_ [14,22,23], but their coordinated regulatory effects with leaf anatomy in regulating intra-leaf CO_2_ diffusion and carbon assimilation have not yet been comprehensively elucidated within contemporary botanical research.

In light of these considerations, some anatomical and biochemical parameters, such as the mesophyll cell wall thickness (*T*_cw_), *S*_m_, *S*_c_, AQPs, CA, Rubisco and ABA, were quantified and systematically examined to delineate the interdependent relationships between leaf structural characteristics and biochemical metabolism in regulating CO₂ diffusion and photosynthetic carbon assimilation capacities against the increased atmospheric N deposition. We propose three scientific hypotheses: (i) the enhancement of intra-leaf CO_2_ diffusion and carbon assimilation capacity by soil N primarily stems from its augmentation in *g*_m_ rather than in *g*_sc_; (ii) the improvement in plant biochemical metabolic capacity constitutes the dominant mechanism through which soil N increases *g*_m_; and (iii) the positive effect of the anatomical structure of leaves on *g*_m_ arises through its indirect promotion of enhanced biochemical metabolic capacity in plants. This methodological framework seeks to establish a theoretical foundation for advancing integrated anatomical and molecular biological investigations into plant photosynthetic carbon assimilation mechanisms.

## Materials and methods

### Materials

The disease-resistant and drought-resistant soybean (*Glycine max* (Linn.) Merr.) Jindou 23 (Guoshendou 2001011) was selected as the experimental material to conduct a pot experiment at the Experimental Station of the College of Resources and Environment, Shanxi Agricultural University, Taigu (112°28′ east longitude, 37°12′ north latitude), Shanxi Province, at 764 m a.s.l. In April 2022, three plump soybean seeds were sown in the topsoil of 18.9-L pots measuring 25.0 cm in height and 31 cm in diameter, each holding 12 L of soil. The concentrations of nitrogen (N), phosphorus (P) and potassium (K) in the potted soil were 0.90 ± 0.03, 2.72 ± 0.43 and 30.31 ± 1.94 g kg^-1^, respectively.

### Experimental design

The soybeans were grown under three nitrogen addition levels of 7.5 g urea m^-2^ (low N addition, LN), 15.0 g urea m^-2^ (medium N addition, MN) and 22.5 g urea m^-2^ (high N addition, HN), respectively, and the treatment without N addition (0 g urea m^-2^) was used as the control (CK). Urea solutions varying in N concentration were sprayed onto the containers during the flowering period on July 5, 2022.The growth cycle of the test plants was from April to August 2022, totaling 138 days. During the experiment, all the plants were maintained outdoors under natural sunlight conditions and were fully watered to eliminate the impact of water deficit. In addition, a solution of total nutrients was applied to the potted plants twice per month to maintain their optimum growth conditions, and the composition and content of the total nutrient solution are listed in Zhu et al. [14]. A 4.5-m long, 3-m wide and 2.1-m high rain shelter covered with a transparent plastic film (99% light transmittance) was established to eliminate the impacts of atmospheric N deposited during natural precipitation, and all the potted plants were placed under the shelter.

### Simultaneous gas exchange and chlorophyll fluorescence measurements

Leaf gas exchange and chlorophyll fluorescence were simultaneously measured in the fully expanded and sun-exposed leaves from 8:00–11:30 each day under a saturated photosynthetic active photon flux density (PPFD) of 1500 µmol m^-2^ s^-1^ with a red:blue light ratio of 90:10 using an open-flow gas exchange system (Li-6400XT; LI-COR Inc., Lincoln, NE, USA) equipped with an integrated fluorescence leaf chamber (Li-6400–40; LI-COR). The leaf temperature and relative humidity (RH) in the leaf chamber were set to 25°C and 60%, the CO_2_ concentration was maintained at 400 µmol CO_2_ mol^-1^ via a CO_2_ mixture, and the flow rate was controlled at 300 μmol s^-1^. The gas exchange parameters, steady-state and maximum fluorescence (*F*_s_ and *F*_m_*′*) of leaves, with a light-saturating pulse of 7800 µmol m^-2^ s^-1^, were recorded via the multiphase flash method [24] after full light adaptation at an intensity of 1500 µmol m^-2^ s^-1^ for 25 ~ 30 minutes.

The actual photochemical efficiency of photosystem II (*Φ*_PS II_) was calculated using the method of Genty et al. [25]:


$$\Phi_{\text{PSII}}=\frac{F_{\text{m}}^{\prime }-F_{\text{s}}}{F_{\text{m}}^{\prime }}$$ (1)

The electron transport rate (*J*_f_, μmol e^-^ m^-2^ s^-1^) was then calculated as follows:


$$J_{\text{f}}=\Phi_{\text{PS}\text{II}}\cdot PPFD\cdot \alpha \beta$$ (2)

where *α* represents the leaf absorptance and *β* represents the distribution of absorbed quanta between PS Ⅱ and PS Ⅰ. In this study, *αβ* was calibrated as the slope of the relationship between the net photosynthetic rate (*A*_n_) and PPFD·*Φ*_PS Ⅱ_/4 using the method of Yin et al. [26], which was obtained from the *A*_n_/PPFD curves under a low O_2_ concentration (< 1%) that was achieved by injecting pure N_2_.

The *g*_m_ was estimated by the ‘variable *J*’ method described in Harley et al. [27]:


$${\text{g}}_{\text{m}}=\frac{A_{\text{n}}}{C_{\text{i}}-\frac{\Gamma^{*}\left(J_{\text{f}}+\text{8}\left(A_{\text{n}}+R_{\text{d}}\right)\right)}{J_{\text{f}}-\text{4}\left(A_{\text{n}}+R_{\text{d}}\right)}}$$ (3)

where *C*_i_ is the intercellular CO_2_ concentration, which is directly obtained from gas exchange measurements, and Г^*^ and *R*_d_ represent the CO_2_ compensation points in the absence of respiration and day respiration, respectively.

The *R*_d_ and apparent CO_2_ photocompensation point (*C*_i_^*^) were determined using the Laisk method [28]. Briefly, three *A*_n_/*C*_i_ curves were measured by varying the CO_2_ concentration from 150 to 40 µmol CO_2_ mol^-1^ under three low PPFDs (150, 100 and 50 µmol m^-2^ s^-1^) and then linearly regressed to intersect at a given point. The intersection points were considered to be *C*_i_^*^ (*x*-axis) and *R*_d_ (*y*-axis) [29,30]. Г^*^ was calculated as follows:


$$\Gamma^{*}=C_{\text{i}}^{*}+\frac{R_{\text{d}}}{{\text{g}}_{\text{m}}}$$ (4)

we used a single set of *C*_i_^*^ (44.09 μmol mol^-1^) and *R*_d_ (0.90 μmol m^-2^ s^-1^) values to calculate Г^*^ values for all growth conditions in this study because they were less affected by environmental changes.

Another important indicator of photosynthetic carbon assimilation, i.e., stomatal conductance to CO_2_ (*g*_sc_, mol CO_2_ m^-2^ s^-1^), is the ratio of stomatal conductance to water (*g*_sw_, mol H_2_O m^-2^ s^-1^) and 1.6, where 1.6 is the ratio of the diffusivities of CO_2_ and water in air [29].

### Determinations of leaf water use efficiency

At the leaf level, the instantaneous and intrinsic water use efficiencies (WUE_ins_ and WUE_int_, respectively) were determined as described in Ouyang et al.[31]:


$${\text{WUE}}_{\text{ins}}=\frac{A_{\text{n}}}{E}$$ (5)

$${\text{WUE}}_{\text{int}}=\frac{A_{\text{n}}}{{\text{g}}_{\text{sw}}}$$ (6)

where *E* is the transpiration rate (mmol H_2_O m^-2^ s^-1^).

### Light and electron microscopy

Three leaf discs (4.0 mm × 1.5 mm) per treatment taken from gas exchange-measured leaves were immediately fixed in FAA (alcohol: formaldehyde: glacial acetic acid = 90: 5: 5) and 2.5% glutaric aldehyde in 0.1 M phosphate buffer (pH = 7.6) at 4 °C and were further processed according to the methods described in Zhu et al. [14].

ImageJ software was used to measure the thicknesses of the leaf (*T*_leaf_, μm) and mesophyll tissue (*T*_m_, μm). The surface areas of mesophyll cells (*S*_m_, μm^2^ μm^-2^) and chloroplasts (*S*_c_, μm^2^ μm^-2^) exposed to the intercellular airspace per unit leaf area were calculated according to the methods of Evans et al. [32] and Syvertsen et al. [33].


$$S_{\text{m}}=\frac{L_{\text{m}}}{W}F$$ (7)

$$S_{\text{c}}=\frac{L_{\text{c}}}{W}F$$ (8)

where i = m, c. *L*_i_ is the total lengths of mesophyll cells (*L*_m_, μm) and chloroplasts (*L*_c_, μm) exposed to the intercellular airspace measured with ImageJ software. *W* (μm) was the cross-sectional width. *F* is the curvature correction factor calculated with the methods of Evans et al. [32]and Thain [34]. In this study, the *F* values for the different treatments were determined to be 1.25 (CK), 1.30 (LN), 1.23 (MN) and 1.27 (HN).

Gas**-** and liquid-phase conductances (*g*_ias_ and *g*_liq_, respectively, m s^-1^) were calculated according to the description in Niinemets and Reichstein [35]:


$${\text{g}}_{\text{ias}}=\frac{D_{\text{a}}\cdot f_{\text{ias}}}{{\Delta L}_{\text{ias}}\cdot \zeta }$$ (9)

$${\text{g}}_{\text{liq}}=\frac{\text{1}}{\left(\frac{\text{1}}{{\text{g}}_{\text{cw}}}+\frac{\text{1}}{{\text{g}}_{\text{pl}}}+\frac{\text{1}}{{\text{g}}_{\text{ct}}}+\frac{\text{1}}{{\text{g}}_{\text{en}}}+\frac{\text{1}}{{\text{g}}_{\text{st}}}\right)S_{\text{c}}}$$ (10)

where *D*_a_ (m^2^ s^-1^) is the diffusion coefficient for CO_2_ in the gas phase (1.68 × 10^−5^ at 30°C) [36]. Δ*L*_ias_ (μm) was taken as half of *T*_m_ [35]. ϛ is the diffusion path tortuosity (m m^-1^), which was equal to 1.57 [33,35]. *f*_ias_ (%) is the fraction of mesophyll volume occupied by the intercellular airspace that was calculated according to Syvertsen et al. [33]. *g*_cw_, *g*_pl_, *g*_ct_, *g*_en_ and *g*_st_ are the partial conductance values for the cell wall, plasmalemma, cytosol, chloroplast envelope and chloroplast stroma (m s^-1^), respectively. The value of 0.0035 m s^-1^ was taken as the value for both *g*_pl_ and *g*_en_ [37], and *g*_cw,_ *g*_ct_ and *g*_st_ were calculated using the method in Niinemets and Reichstein [35] as follows:


$${\text{g}}_{\text{i}}=\frac{r_{\text{f},\text{i}}\cdot D_{\text{w}}\cdot p_{\text{i}}}{\Delta L_{\text{i}}}$$ (11)

where ΔLi (m) represents the diffusion distance within the relevant diffusion component, pi (m³·m ⁻ ³) denotes its effective porosity, and Dw corresponds to the aqueous-phase diffusion coefficient for CO₂ (2.03 × 10 ⁻ ⁹ m²·s ⁻ ¹ at 30°C). In this analysis, Δ*L*_i_ was set to 1.2 × 10^−7^ (for Δ*L*_cw_), 2.1 × 10^−7^ (for Δ*L*_ct_) and 1.6 × 10^−6^ (for Δ*L*_st_) according to these values for *Phaseolus vulgaris* L. [35]. The dimensionless coefficient *r*_f_ accounts for the decrease in diffusion conductance in the cell wall, cytosol and chloroplast stroma. *r*_f,i_ values for the cytosol (*r*_f,ct_) and chloroplast stroma (*r*_f,st_) were estimated to be 0.294, and *r*_f,cw_ = 1 for the cell wall [36]. The cell wall porosity (*p*_cw_) varied with the *T*_cw_, according to Tosens et al. [37] (*p*_cw_ = −0.3733 × *T*_cw_ + 0.3378), and *p*_i_ was set to 1.0 for the cytosol (*p*_ct_) and stroma (*p*_st_) (Nobel, 1991). Conductance in units of m s ⁻ ¹ can be converted to molar units via the formula: g (mol m ⁻ ² s ⁻ ¹) = g (m s ⁻ ¹) × 44.6 × [273.16/ (273.16 + *T*_leaf_)] × (P/ 101.325), with *T*_leaf_ defined as leaf temperature (°C) and P (Pa) as air pressure.

The stomatal pore length (PL, μm) and width (PW, μm) (i.e., the major and minor axes of an ellipse) were analyzed with ImageJ software to determine the stomatal opening status at the stoma center (*SS*, μm^2^) [11].:


$$\text{SS}=\frac{\pi \cdot \text{PL}\cdot \text{PW}}{\text{4}}$$ (12)

### Measurements of related biochemical parameters

The biochemical parameters related to CO_2_ diffusion, such as AQPs, the CA and Rubisco contents and activities and the ABA content, were measured via enzyme-linked immunosorbent assay (ELISA) in three fresh leaves per sample in each treatment according to Maeda et al. [38], as described in detail in Zhu et al. [14].

### Statistical analysis

To ensure data validity, normality and homogeneity of variance were verified using SPSS 17.0 (SPSS Inc., Chicago, IL, USA) prior to further statistical procedures. Correlations between CO_2_ diffusion conductances (*g*_m_, *g*_sc_ and *g*_m_/*g*_sc_) and WUE (WUEins and WUEint), and between gliq and CA activity, were then executed. Mean values across different treatments were compared using the LSD multiple comparison test at P < 0.05.Principal Component Analysis (PCA) was performed using standardized anatomical and biochemical traits to identify the major dimensions of variation under nitrogen treatments. Additionally, partial correlation analysis was conducted to examine the direct relationships between biochemical and physiological parameters (e.g., CA activity vs. WUE_int_ or *g*_m_/*g*_sc_) while controlling for the effects of nitrogen level and aquaporin activity.

## Results

### Effects of N addition on *g*_m_ and *g*_sc_

**Fig 1:**
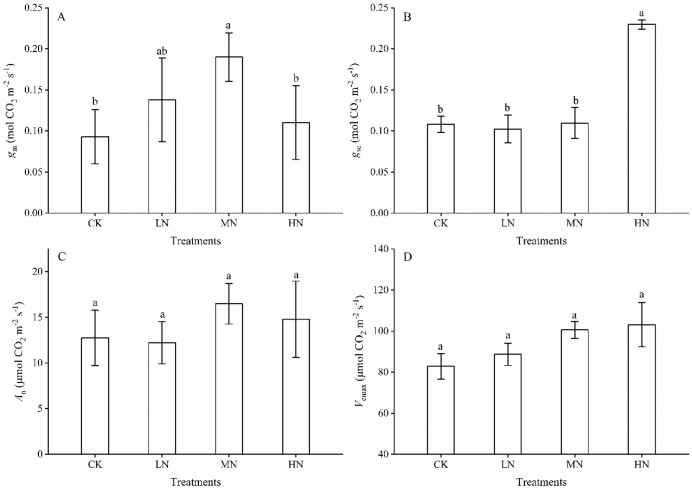
Changes in *g*_m_, *g*_sc_, *A*_n_ and *V*_cmax_ during soil N addition. The error bars represent the standard error of all measurements (n = 3). Different letters indicate significant differences among the groups (*P* < 0.05). *g*_sc_, stomatal conductance; *g*_m_, mesophyll conductance; *A*_n_, net photosynthetic rate; *V*_cmax_, maximum carboxylation rate. CK, control; LN, low N addition; MN, medium N addition; HN, high N addition. The same applies below.

Different effects of the intensity of soil N addition on intra-leaf CO_2_ diffusion were clearly manifested through corresponding variations in *g*_m_ and *g*_sc_ (Fig 1A and B). With the increase in N addition intensity, *g*_m_ showed a fluctuating trend of first increased significantly from 0.09 mol CO_2_ m^-2^ s^-1^ (0 g urea m^-2^ N addition, CK) to 0.19 mol CO_2_ m^-2^ s^-1^ (15.0 g urea m^-2^ N addition, MN) and then decreased to 0.11 mol CO_2_ m^-2^ s^-1^ (22.5 g urea m^-2^ N addition, HN) (*P* < 0.05), but *g*_sc_ did not change overall, except for a significant increase from 0.11 to 0.23 mol CO_2_ m^-2^ s^-1^ under HN (*P* < 0.05). Thus, the different effects of soil N addition on intra-leaf CO_2_ diffusion in soybean were clearly due mainly to its regulation of *g*_m_.

### Response of CO_2_ assimilation capacity

Although soil N addition had a significant positive effect on *g*_m_, it did not have a statistically significant effect on the net photosynthetic or maximum carboxylation rates of CO_2_ (*A*_n_ and *V*_cmax_) (*P* < 0.05; Fig 1C and D). Nevertheless, both *A*_n_ and *V*_cmax_ in N-addition plants were still greater than those in non-N-addition plants, with the mean values in N-addition plants being 14.5 and 97.5 μmol CO_2_ m^-2^ s^-1^, respectively.

### Effects of N addition on leaf water use capacity

**Fig 2:**
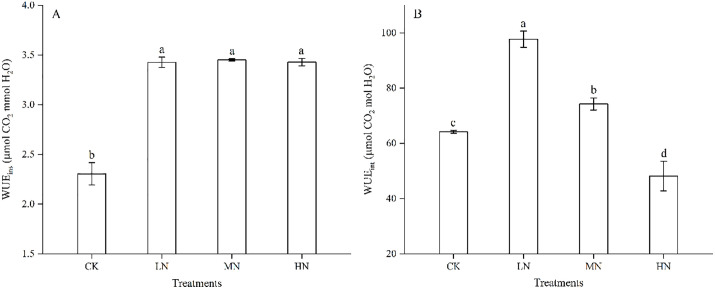
Changes in WUE_ins_, WUE_int_, *E* and *g*_sw_ during soil N addition. WUE_ins_, instantaneous water use efficiency; WUE_int_, intrinsic water use efficiency.

Soil N addition had a significant effect on leaf water use capacity, with WUE_ins_ being greater in N-addition plants than in the CK (Fig 2A), whereas WUE_int_ decreased with increasing N addition from 7.5 to 22.5 g urea m^-2^ (Fig 2B). The different effects of N addition on WUE_ins_ and WUE_int_ should be attributable primarily to the physiological regulation of *g*_sc_ (Fig 1B), as evidenced by the fundamental relationships of WUE_ins_ = *A*_n_/*E* and WUE_ins_ = *A*_n_/*g*_sw_ (*g*_sw_ = 1.6*g*_sc_), where *g*_sw_ denoted *E*.

### Relationships between CO_2_ diffusion conductance and water use efficiency

**Fig 3:**
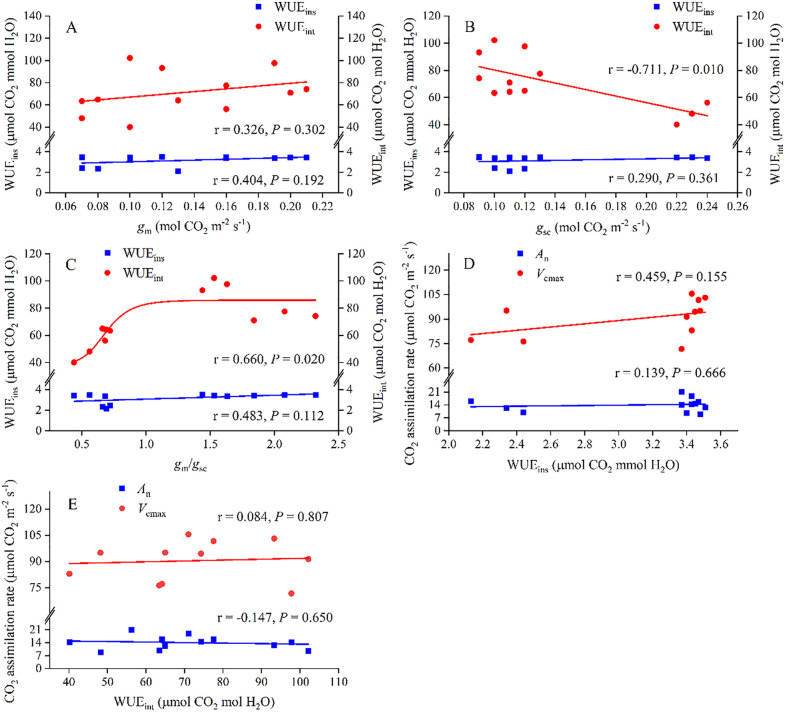
Relationships between *g*_m_, *g*_sc_ or *g*_m_/*g*_sc_ and WUE_ins_ and WUE_int_ and CO_2_ assimilation rates (*A*_n_ and *V*_cmax_) during soil N addition.

The correlations between water use efficiency (WUE_ins_ and WUE_int_) and CO_2_ diffusion conductance (*g*_m_, *g*_sc_ and *g*_m_/*g*_sc_) and CO_2_ assimilation parameters (*A*_n_ and *V*_cmax_) were quantified in Fig 3. WUE_int_ showed a highly significant negative correlation with *g*_sc_ (*P* < 0.01) and a positive correlation with *g*_m_/*g*_sc_ (*P* < 0.05), whereas the correlations between WUE_ins_ and *g*_sc_ and *g*_m_/*g*_sc_ were not significant. Water use efficiency did not significantly differ with respect to *g*_m_ or *A*_n_ and *V*_cmax_. Hence, the pronounced influence of WUE_int_ on the intraleaf CO_2_ diffusion capacity was principally attributable to its regulatory control over *g*_sc_ and *g*_m_/*g*_sc_.

### Changes in leaf anatomical characteristics

#### Leaf anatomical traits.

**Fig 4:**
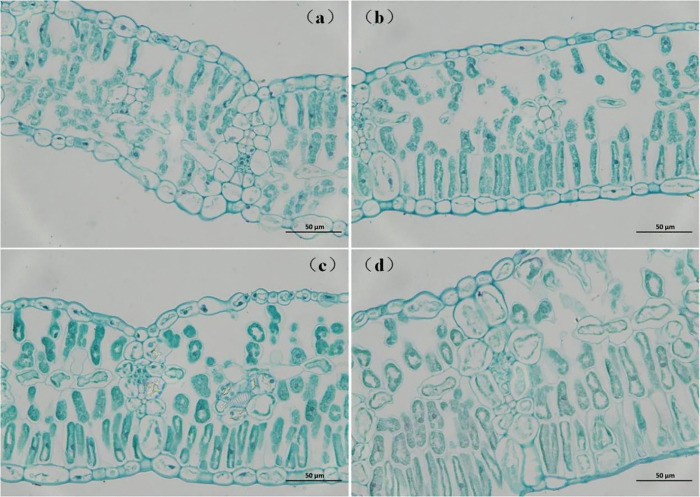
Light micrographs of transverse sections of control and N-addition-treated leaves. All the leaf cross-sections were observed at 400 × magnification. Scale bar = 50 μm. (a) CK, control; (b) LN, low N addition; (c) MN, medium N addition; (d) HN, high N addition. The same applies below.

N addition improved leaf morphological structure by improving the arrangement of palisade and spongy tissues (Fig 4I). Compared with the control group, mesophyll cells presented a more turgid morphology and more orderly arrangement with enlarged intercellular spaces following soil N addition. These structural modifications became increasingly pronounced with increasing N addition.

Quantitative anatomical analysis revealed different responses to N addition in terms of leaf, mesophyll cell and cell wall thicknesses (i.e., *T*_leaf_, *T*_m_, and *T*_cw_) (Table 1). Specifically, both *T*_leaf_ and *T*_m_ were positively correlated with N addition, whereas *T*_cw_ was inversely related. Collectively, *S*_m_, *V*_m_ and *V*_c_ exhibited proportional increases with N supplying, contrasting with *S*_c_ which displayed a progressive reduction under elevated N additions. The *SS* was increased by soil N nutrient and it furtherly increased with N supplying, especially a significant increase under HN condition (*P* < 0.05). Overall, *T*_cw_, *S*_m_ and *S*_c_ exhibited the most pronounced alterations under soil nitrogen addition rates of 7.5 and 15.0 g urea m^-2^, demonstrating a strongest structural responsiveness at these specific application intensities.

**Table 1:** Values of leaf anatomical traits under different soil N addition treatments. All the data are the means ± SEs (n = 3). Different lowercase letters (a, b, c) indicate significant differences at *P* < 0.05. *T*_leaf_, total leaf thickness; *T*_m_, mesophyll tissue thickness; *T*_cw_, cell wall thickness; *SS*, stomatal opening status; *S*_m_, surface area of mesophyll cells exposed to intercellular space per unit leaf area; *S*_c_, chloroplast surface facing the intercellular space per unit leaf area; *V*_m_, volume of mesophyll cells per unit leaf area; *V*_c_, chloroplast volume per unit leaf area; *g*_ias_, gas-phase conductance to CO_2_; *g*_liq_, liquid-phase conductance to CO_2_.

Treatments	*T*_leaf_ (μm)	*T*_m_ (μm)	*T*_cw_ (μm)	SS (μm^2^)	*S*_m_ (μm^2^)	*S*_c_ (μm^2^)	*V*_m_ (μm^3^)	*V*_c_ (μm^3^)	*g*_ias_ (mol CO_2_ m^-2^ s^-1^)		*g*_liq_ (10^−3^ mol CO_2_ m^-2^ s^-1^)
CK	131.3 ± 8.9^c^	104.5 ± 12.4^c^	0.36 ± 0.04^a^	11.4 ± 2.0^b^	6.52 ± 0.24^c^	3.69 ± 0.58^a^	21.1 ± 3.0^b^	2.76 ± 0.49^a^		8.95 ± 1.10^a^	3.10 ± 0.50^a^
LN	156.5 ± 10.2^b^	128.4 ± 12.4^b^	0.25 ± 0.03^b^	8.8 ± 0.1^b^	13.48 ± 2.47^b^	3.74 ± 0.91^a^	40.1 ± 4.0^b^	2.87 ± 0.28^a^		7.27 ± 0.69^b^	3.13 ± 0.80^a^
MN	174.3 ± 12.9^b^	140.0 ± 9.1^b^	0.28 ± 0.04^ab^	11.8 ± 0.9^b^	16.87 ± 0.08^a^	2.71 ± 0.50^a^	38.8 ± 9.4^b^	2.94 ± 0.22^a^		6.68 ± 0.43^b^	4.27 ± 0.84^a^
HN	225.6 ± 16.5^a^	189.1 ± 13.2^a^	0.35 ± 0.09^a^	18.5 ± 3.0^a^	13.66 ± 0.55^b^	3.28 ± 0.61^a^	79.5 ± 17.8^a^	3.04 ± 0.08^a^		4.89 ± 0.29^c^	3.54 ± 0.71^a^

### Gas- and liquid-phase conductances

**Fig 5:**
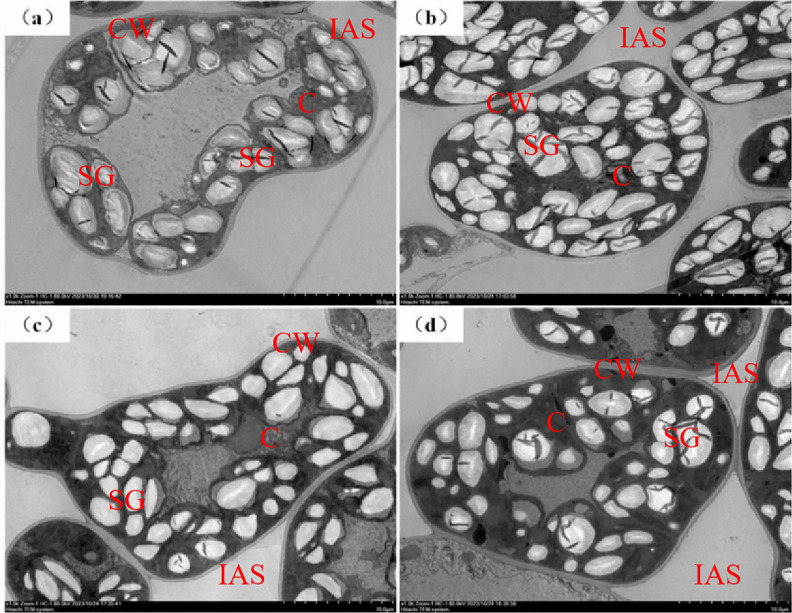
Transmission electron microscope images of the control and N-addition-treated leaves. Scale bar = 10 μm. C, chloroplast; CW, mesophyll cell wall; SG, starch granule; IAS, intercellular airspace.

**Fig 6:**
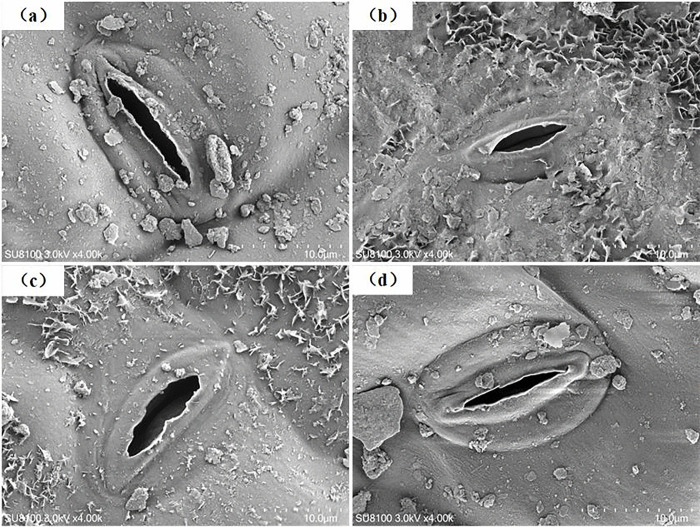
Scanning electron microscopy (SEM) images of stoma from the control group and N-addition-treated leaves. Scale bar = 10 μm.

The CO_2_ gas-phase diffusional capacity from substomatal cavities to the outer surface of cell walls was weakened by soil N supplementation, with *g*_ias_ values decreasing from 8.95 to 4.89 mol CO_2_ m^-2^ s^-1^ with N addition (Table 1). In contrast to *g*_ias_, *g*_liq_ was increased by N addition overall, with no significant difference (*P* < 0.05), especially under the addition level of 15.0 g urea m^-2^, indicating that the CO_2_ liquid-phase diffusional capacity from the outer surface of cell walls to chloroplasts was greatly strengthened by 15.0 g of urea m^-2^ N addition (Fig 5, 6).

### Changes in leaf biochemical parameters

Leaf biochemical parameters showed a dependence on N addition levels in soybean (Table 2). The contents of AQPs and CA and the activities of CA and Rubisco were significantly increased by soil N addition (Table 2). A significant statistical difference was found in CA content and the activities of CA and Rubisco (*P* < 0.05, Table 2). Differed from AQPs, CA and Rubisco, the content of ABA decreased overall, where it was significantly decreased by the HN level (*P* < 0.05).

**Table 2:** Values of the key biochemical traits in the different soil N addition treatments. All data are mean ± SE (n = 3). Different lowercase letters (a, b, c) indicate significant differences at *P* < 0.05. AQPs, aquaporins; CA, carbonic anhydrase; Rubisco, ribulose-1,5-bishosphate carboxylase; ABA, abscisic acid.

Treatments	AQPs		CA		Rubisco		ABA
	content (ng L-1)	activity (U L-1)	content (ng L-1)	activity (U L-1)	content (ng L-1)	activity (U L-1)	content (ng g-1)
CK	18.6 ± 0.4^b^	643.7 ± 27.8^a^	51.0 ± 0.4^a^	198.8 ± 3.4^d^	142.1 ± 7.2^a^	690.5 ± 4.5^c^	316.5 ± 5.7^a^
LN	22.0 ± 0.7^a^	604.0 ± 13.0^b^	52.6 ± 3.8^a^	214.7 ± 3.3^c^	153.3 ± 8.6^a^	807.1 ± 3.4^b^	310.1 ± 6.1^a^
MN	22.7 ± 0.9^a^	644.7 ± 44.5^a^	53.8 ± 2.0^a^	223.8 ± 4.5^b^	146.9 ± 13.8^a^	836.8 ± 15.5^a^	313.6 ± 4.2^a^
HN	22.1 ± 0.2^a^	678.3 ± 8.0^a^	53.7 ± 0.4^a^	238.3 ± 2.6^a^	156.2 ± 6.1^a^	807.4 ± 1.2^b^	286.3 ± 7.7^b^

## Discussion

Nitrogen (N) addition positively affected plant physiological functions [9,39,40], but its positive effects were dependent on the N addition intensity [23,41,42]. In the present study, the net photosynthetic rate (*A*_n_) of soybean leaf presented an N response coherent with *g*_m_ rather than *g*_sc_, with *A*_n_ and *g*_m_ increasing with N supplying from 7.5 to 15.0 g urea m^-2^ and then decreasing from 15.0 to 22.5 g urea m^-2^ (Fig 1A and C). The *g*_sc_ did not exhibit any difference with N addition, except for a transient increase at the HN level (Fig 1B). *T*_cw_, *S*_m_ and *S*_c_ had been further highlighted as the most important structural components determining *g*_m_[11,19,36].We systematically investigated the mechanisms by which soil N enhanced CO₂ diffusion from these dual perspectives. Although increased *S*_m_ under N addition (Table 1) exerted a positive effect on CO₂ liquid-phase diffusion capacity, the observed reductions in *S*_c_ following N application diminished the contact area between CO₂ and chloroplast carboxylation sites and made a negative effect on *g*_m_ increase. In addition, the decreased *T*_cw_ (Table 1) would decrease cellulose and pectin content within cell walls and consequently reduce cell walls’ elasticity or rigidity and set a limitation for maximum *g*_m_ [43, 44], thereby increasing resistance to CO₂ diffusion through both cell walls and chloroplast membranes, as *T*_cw_ was suggested to be related to the bulk modulus of elasticity (ɛ), which represented the elasticity or rigidity of leaf tissues [45]. These results indicate that N-driven enhancements in photosynthetic carbon assimilation occurred primarily through mesophyll-level processes rather than stomatal adjustments. Although N addition induced anatomical alterations—increasing *S*_m_ but reducing *S*_c_ and *T*_cw_, which theoretically should constrain *g*_m_—both *g*_m_ and carbon assimilation parameters (i.e., *A*_n_ and *V*_cmax_) increased in response to N addition (Fig 1). This contrast suggests that the positive effects of N supplementation were predominantly mediated by mechanisms beyond structural modifications, specifically through the enhancement of biochemical metabolism.

**Fig 7:**
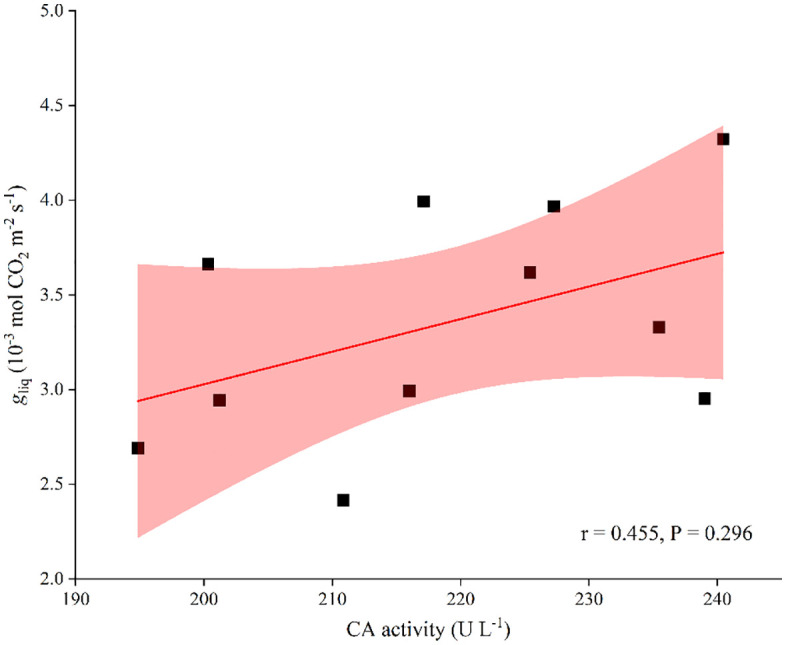
Correlation between CA activity and *g*_liq_ in soybean. Regression coefficients and significance are shown when *P <* 0.05. CA, carbonic anhydrase; *g*_liq_, liquid-phase CO_2_ diffusion conductance.

**Fig 8:**
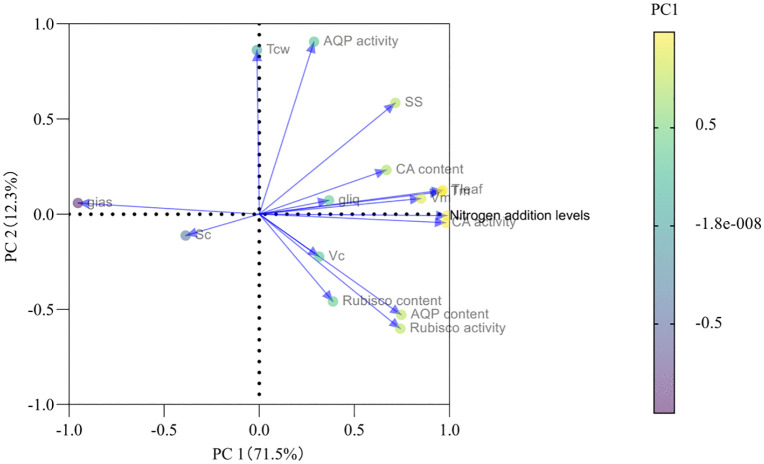
The PCA loadings plot between biochemical and anatomical parmeters. AQPs, aquaporins; CA, carbonic anhydrase; Rubisco, ribulose-1,5-bisphosphate carboxylase; *T*_cw_,cellwall thickness; *S*_c_, stomatal conductance; *SS*, stomatal opening; *V*_m_, volume of mesophyll cells per unit leaf area; *V*_c_, chloroplast volume per unit leaf area; *g*_ias_, gas-phase conductance to CO_2_; *g*_liq_, liquid-phase conductance to CO_2_.

In contrast to the adverse effects induced by leaf anatomical modifications, N addition significantly increased both CA and Rubisco contents and activity (*P* < 0.05; Table 2). This would markedly improve the solubility of CO_2_ in the cell matrix [7,19] and accelerate the conversion of gaseous CO₂ to HCO₃⁻ [17,21] and carboxylation efficiency, thereby leading to an increase in *g*_liq_ (Table 1), which was supported by the positive correlation between CA activity and *g*_liq_ (Fig 7). The PCA loadings plot (Fig 8) quantitatively illustrates the contribution of each measured variable to the principal components. Biochemical activity parameters, specifically CA activity and Rubisco activity, exhibited the strongest loadings on PC1, which alone explained 71.5% of the total variance. Their vectors were longest and aligned almost exclusively with the positive direction of PC1, identifying them as the dominant drivers of the physiological response to nitrogen supply. In contrast, anatomical parameters (e.g., *T*_CW_, *SS*) and aquaporin activity (AQP activity) showed weaker associations with PC1, indicating their secondary role in this process.

We have revealed the negative correlations between ABA and *g*_sc_ and *g*_m_ in our early study of Zhu et al. [14]. The decrease in leaf ABA content with N addition (Table 2) potentially alleviated its inhibitory effects on stomatal opening and AQPs expression [46,47]. Furthermore, the reduction in ABA might also reflect a shift in hormonal balance, favouring growth-promoting hormones such as cytokinin (CTK) under HN conditions, and this hypothesis requires further validation through hormone profiling. Consequently, the enhancement of biochemical metabolism rather than the modifications in the leaf anatomical structure constituted the predominant mechanism by which N supplementation facilitated CO_2_ diffusion and carbon assimilation in soybean, which was also suggested by the statistical results, in which the leaf anatomical parameters did not significantly differ among the treatments (*P* > 0.05; Table 1), whereas the biochemical parameters, except ABA, significantly varied with N supply (*P* < 0.05; Table 2).

Notably, the dominant mechanism of biochemical metabolism was reinforced by improved leaf water-use efficiency (WUE), as we have suggested a significant correlation between WUE and *g*_sc_/*g*_m_, i.e., in Zhu et al. [14]. A positive correlation and a significant negative correlation were also found between WUE_int_ and *g*ₘ/*g*_sc_ (*P* < 0.05, Fig 3C) and between WUE_int_ and *g*_sc_ (*P* < 0.01, Fig 3B). This further substantiated the regulatory role of WUE in the intra-leaf CO₂ diffusion capacity, with the underlying mechanism likely originating from its influence on Rubisco activity [44,48,49]. Rubisco activity significantly increased (Table 2), which greatly increased the CO_2_ carboxylation capacity by increasing *V*_cmax_ (Fig 1D) and WUE_int_ (the ratio of *A*_n_ to *g*_sc_, Fig 2B). The increased WUE_int_ would inevitably lead to increased *g*_m_ and carbon assimilation, as increased *g*_m_ increased WUE_int_ [4,50]. These findings suggested that plants under N addition preferentially optimize mesophyll CO_2_ utilization over stomatal CO_2_ uptake to balance carbon assimilation with water conservation. To quantitatively evaluate the relationships between biochemical enhancement and water relation parameters, we performed partial correlation analyses. The results revealed that aquaporin (AQPs) activity showed a strong direct correlation with the stomatal-to-mesophyll conductance ratio (*g*_m_/*g*_sc_; *r* = 0.866, *P* < 0.001), indicating a primary role of water relations in coordinating diffusive conductances. Meanwhile, carbonic anhydrase (CA) activity did not exhibit a significant direct effect on intrinsic water use efficiency (WUE_int_) or *g*_m_/*g*_sc_ after controlling for nitrogen level and AQPs activity (*P* > 0.05), and we would further explore their mechanisms in future studies (Table 3). The lack of correlation between WUE_int_ and *g*_m_ suggested that *g*_m_ was influenced by multiple interacting factors, including anatomical adaptations and biochemical adjustments, rather than a single dominant pathway.

**Table 3:** Relationships between CA activity, AQPs activity, and plant water status indicators, controlling for specific variables.

Relationship	Controlled Variable	Partial r	P-value	Interpretation
CA activity vs. *g*_m_/*g*_sc_	AQPs activity	0.010	0.977	No direct relationship
CA activity vs. WUE_int_	Nitrogen level	0.220	0.516	No direct relationship
AQPs activity vs. *g*_m_/*g*_sc_	None	0.866	<0.001	Strong direct relationship

Additionally, the observed *g*_m_ dynamics in this study aligned with previous studies showing that optimal N increased the intra-leaf CO_2_ diffusion efficiency by improving biochemical and structural traits, whereas excessive N disrupted these mechanisms through metabolic imbalances [11,13]. Furthermore, excessive N induced leaf thickening and starch accumulation (Fig 7), likely due to disrupted carbon-N partitioning. These structural changes reduced the intercellular airspace and limit the gas-phase CO_2_ diffusion process, as gias decreased from 8.95 to 4.89 mol CO_2_ m^-2^ s^-1^ under HN (Table 1). These findings underscore the delicate trade-off whereby moderate N enhanced photosynthetic capacity through biochemical activation, whereas supra-optimal N imposed physical barriers and metabolic toxicity.

## Conclusion

Soil nitrogen (N) had a positive effect on intra-leaf CO_2_ diffusion and carbon assimilation in soybean, with *g*_m_ and *A*_n_ (net photosynthetic rate) significantly increasing with N supplying from 7.5 to 15.0 g urea m^-2^, whereas *g*_sc_ showed no significant N dependence. A high N addition of 22.5 g urea m^-2^ had a negative effect on *g*_m_ and *A*_n_. The enhanced intra-leaf CO₂ diffusion capacity induced by N application was principally attributable to its augmentative effect on *g*_m_. The improved leaf structure and enhanced biochemical metabolism constituted the primary mechanisms through which increased soil N elevated intra-leaf CO_2_ diffusion and carbon assimilation, with biochemical metabolic enhancement acting as the dominant driver. In addition, the improvement in water use efficiency, with WUE_ins_ significantly increasing while WUE_int_ initially increases but then decreases, might have a great positive effect on *g*_m_ from the perspective of water dynamics.

## Supporting information

S1 File Raw data of photosynthetic fluorescence parameters and leaf anatomical structures under nitrogen treatments. (XLSX)
